# Bioenergy-producing two-stage septic tank and floating wetland for onsite wastewater treatment: Circuit connection and external aeration

**DOI:** 10.1016/j.jenvman.2024.121011

**Published:** 2024-05

**Authors:** Tanveer Saeed, Abdullah Al-Muyeed, Takrim Zaman, Mehedi Hasan, Tanvir Ahmed

**Affiliations:** a Department of Civil Engineering, University of Asia Pacific, Dhaka, 1205, Bangladesh; b Institute of Energy, Environment, Research and Development (IEERD), University of Asia Pacific, Dhaka, 1205, Bangladesh; c CWIS-FSM Support Cell, Department of Public Health Engineering, Government of the People's Republic of Bangladesh, Dhaka, 1000, Bangladesh; d ITN-BUET Centre for Water Supply and Waste Management, Bangladesh University of Engineering and Technology, Dhaka, 1000, Bangladesh; e Department of Civil Engineering, Bangladesh University of Engineering and Technology, Dhaka, 1000, Bangladesh

**Keywords:** Decentralized wastewater treatment, Electrical conductivity, Energy, External air supply, Removal pathways

## Abstract

This study designed a two-stage, electrode-integrated septic tank-floating wetland system and assessed their pollutant removal performances under variable operational conditions. The two-stage system achieved mean organic, nitrogen, phosphorus, and coliform removal percentages of 99, 78, 99, and 97%, respectively, throughout the experimental run. The mean metals (chromium, cadmium, nickel, copper, zinc, lead, iron, and manganese) removal percentages ranged between 81 and 98%. Accumulated sludge, filler media, and the hanging root mass contributed to pollutant removals by supporting physicochemical and biological pathways. The mean effluent organic concentration and coliform number across the two-stage system were 20 mg/L and 1682 CFU/100 mL, respectively, during the closed-circuit protocol, which was beneath the open-circuit-based performance profiles, i.e., 32 mg/L and 2860 CFU/100 mL, respectively. Effluent organic, nitrogen, phosphorus, metals, and coliform number ranges across the two-stage system were 9–17 mg/L, 13–24 mg/L, 1–1.5 mg/L, 0.001–0.2 mg/L, and 1410–2270 CFU/100 mL, respectively during intermittent and continuous aeration periods. The air supply rate differences influenced pollutant removal depending on the associated removal mechanisms. The non-aeration phase produced higher effluent pollutant concentrations than the aeration periods-based profiles. The overall mean power density production of the septic tank ranged between 107 and 596 mW/m^3^; 110 and 355 mW/m^3^ with the floating wetland. The bioenergy production capacity of the septic tank was positively correlated to external air supply rates. This study demonstrates the potential application of the novel bioenergy-producing septic tank-floating wetland system for wastewater treatment in decentralized areas.

## Highlights

• A bioenergy-producing two-stage septic tank-floating wetland was designed.
• A mean of 99% COD, 78% TN, and 99% TP removal was achieved.
• The mean metals removal percentages ranged between 81 and 98%.
• Closed circuit and aeration protocols improved operational performance.
• The mean power density production ranges were 107 and 596 mW/m^3^.

## Graphical abstract

**Figure:**
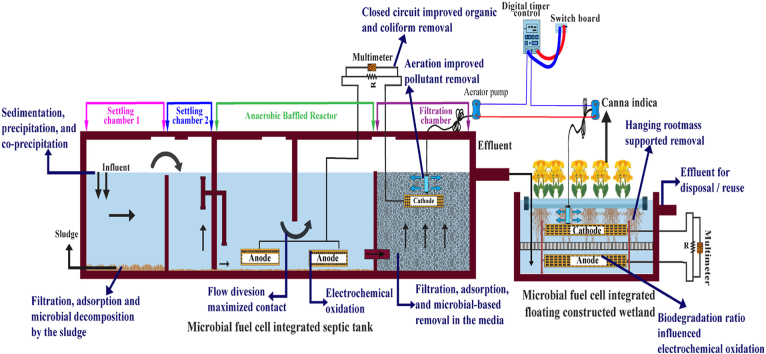

## 1 Introduction

Septic tanks often receive particular attention for onsite wastewater management in decentralized rural and suburban areas (Daija et al., 2016; Sánchez et al., 2023; Sharma et al., 2014). Organics and solid removals in septic tanks rely upon physicochemical pathways (filtration and adsorption) and partial microbial decomposition induced by the accumulated sludge (Adhikari and Lohani, 2019; Nasr and Mikhaeil, 2013; Pishgar et al., 2021; Saeed et al., 2021a). However, inefficient nutrient removal is a major operational drawback of the septic tanks, primarily due to the lack of additional contact with the settled sludge biomass (Saeed et al., 2021a). Several research studies modified the structural design of the septic tanks and achieved notable pollutant removals, primarily due to maximizing the contact between septic tank components (i.e., settled sludge, biomass, and filler media) and wastewater pollutant (Daija et al., 2016; Koottatep et al., 2020; Nasr and Mikhaeil, 2013; Saeed et al., 2021a). More research studies are required on structural modification to optimize the integrated contact between the septic tank components and wastewater pollutant that might improve operational efficiencies (Haydar et al., 2018; Singh et al., 2019).

Constructed wetlands, which depend upon its components (plant, media, and microbial population) for removing wastewater pollutant have also been employed to treat septic tank effluent (Cui et al., 2012; Jácome et al., 2016; Jamwal et al., 2021; Saeed and Sun, 2017). Moreira and Dias (2020) reviewed pollutant removal performances of the combined septic tank-constructed wetlands employed worldwide to treat rural wastewater and reported organic, nitrogen, and phosphorus removal percentages of 89%, 61%, and 67%, respectively. Therefore, integrating wetlands appeared to counterbalance the inefficiencies (i.e., partial pollutant removals) of the septic tank systems, which might be improved by implementing intensified wetlands. Microbial fuel cell or electrode-based constructed wetlands are a notable example of such intensified technologies with unique capacities of concurrent wastewater treatment and bioenergy generation (Gupta et al., 2021; Saeed et al., 2021c). The integrated electrodes support electrochemical-based removal pathways, along with electrochemically inactive routes in such intensified wetlands, resulting in better operational performances (Gupta et al., 2021; Saeed et al., 2022a; Srivastava et al., 2020). These advantages of the electrochemical technologies might be extended to the structurally modified septic tanks or the integrated septic tank-wetland systems to improve overall operational performances and produce effluent quality for safe disposal or reuse (along with bioenergy production) in decentralized areas but have not been reported to date. Effluent reuse is often considered a primary objective of an onsite or decentralized wastewater treatment system (Sánchez et al., 2023).

Maintaining an aerobic environment in the cathode compartment and developing a stratified redox gradient between the electrodes are two performance-related prerequisites with the bioenergy-producing wetlands (Guadarrama-Pérez et al., 2019; Gupta et al., 2021). In the absence of a naturally developing redox gradient within the electrodes of bioenergy-producing wetlands, the operational prerequisites were fulfilled through external air supply (Oon et al., 2017; Saeed et al., 2023b; Srivastava et al., 2017). Due to oxygen scarcity in a normal (without electrodes) septic tank (Pishgar et al., 2021), and the requirement to maintain a stratified redox gradient between the electrodes of the electrochemical technologies (Gupta et al., 2021), external aeration might be an effective approach for intensifying electrochemical-based pollutant removal (with electrodes-based septic tanks) and producing higher effluent quality. Such a hypothesis has to be validated by designing an experimental protocol with an electrode-based septic tank integrated with external aeration.

Metals in wastewater, even in minor concentrations, could threaten the receiving aquatic environment (Ayaz et al., 2020). The review studies on decentralized wastewater treatment technologies employed worldwide were primarily inclined towards removal performance assessment with the common pollutant, i.e., organic, nutrient, solids, and coliform (Geetha Varma et al., 2022; Moreira and Dias, 2020; Saeed and Sun, 2017). Due to data scarcity, the probable metals removal mechanisms in septic tanks and constructed wetlands (when employed to polish septic tank effluent) and performance influencing factors have not clearly been understood to date. Hence, scientific documentation on metals removal routes in decentralized systems is necessary for better design and environmental sustainability.

This study developed a multi-compartment septic tank followed by a floating wetland for sewage treatment to abridge some of the knowledge gaps (as mentioned in the previous paragraphs) in this field. Both treatment units were integrated with electrodes. The two-stage system was operated under variable operational protocols such as circuit connections (closed and open circuit), aeration (intermittent and continuous), and non-aeration strategies. The main objectives of this research were two-folds: (a) to assess the removal routes of pollutant and their interaction with the components (sludge, media, and plant) of the two-stage system; and (b) to investigate the impact of the operational variables (i.e., circuit connections and aeration) on pollutant removal and bioenergy production.

## 2 Materials and method

### 2.1 Construction of the bioreactor septic tank and floating wetland

**Fig. 1:**
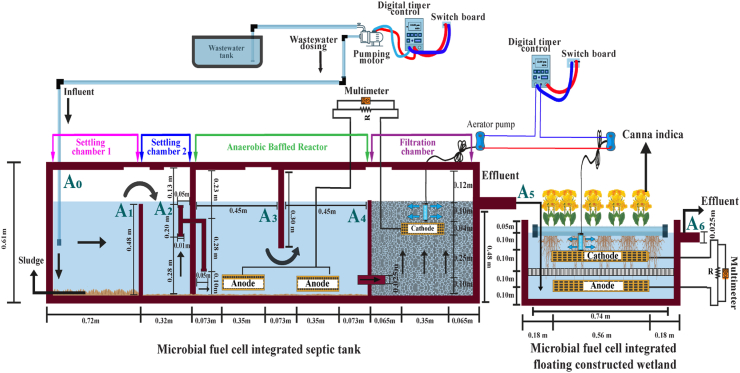
Schematic diagram of the electrode-integrated two-stage septic tank-floating wetland. The symbol A_0_ indicates influent wastewater. The symbols A_1_, A_2_, A_3_, A_4_, A_5_, and A_6_ represent wastewater collection points from settling chamber 1, settling chamber 2, the first compartment of the ABR, the second compartment of the ABR, filtration chamber, and floating wetland, respectively.

A septic tank and a floating wetland were built with steel plates at the University of Asia Pacific, Dhaka, Bangladesh. The schematic diagrams of such treatment units are presented in Fig. 1. The length, width, and height of the septic tank were 2.44, 0.61, and 0.61 m, respectively. The septic tank was built as an air-tight unit (to prevent atmospheric oxygen diffusion) and divided into four chambers: two settling chambers, followed by an ABR, and a filtration unit as the final compartment. The first and second settling chamber was divided by a steel plate (of 0.48 m height) that allowed overflowing of the wastewater from the first to second settling chamber; the effective volume of the first and second settling chambers were 0.22 and 0.10 m^3^, respectively.

An ABR followed the second settling chamber; the effective volume of the ABR compartment was 0.27 m^3^. A solid steel wall completely separated the second settling chamber and the ABR; an inverted L-shaped pipe was integrated into this solid steel wall. The wastewater was collected at a depth of 0.28 m (measured from the bottom of the septic tank) in the second settling chamber. The collected wastewater was transferred to the inverted L-shaped pipe and disposed of at a depth of 0.10 m (measured from the bottom of the septic tank) in the ABR. A steel plate or baffle wall (0.30 m height) was integrated into the ABR at a horizontal distance of 0.45 m from the solid wall separating the second settling chamber and the ABR. Hence, the ABR was divided into two compartments (by the baffle wall) for intercepting the influent and achieving an upflow-downflow pattern (a typical characteristic of an ABR chamber) while flowing towards the outlet.

The ABR was followed by a filtration chamber that was filled with stone materials. The size of such stone materials ranged between 4 and 10 mm, whereas the porosity was 28%. The stone materials achieved a depth of 0.49 m in the filtration chamber; the effective volume of the filtration chamber was 0.14 m^3^. The filtration chamber received ABR effluent through a wastewater collection valve located at a depth of 0.10 m from the bottom of the septic tank. Wastewater flowed vertically upward through the packed stone media (in the filtration chamber) towards the effluent collection valve located at a depth of 0.48 m measured from the bottom of the tank. The effluent of the filtration chamber was the final output produced from the septic tank.

Two steel mesh-charcoal-based anode electrodes were placed on the bottom portion of both ABR compartments that were divided by the baffle wall. A steel mesh-charcoal-built cathode electrode was placed at 0.14 m depth beneath the stone media surface in the filtration chamber and was connected to both anode electrodes through external electrical circuits, an 820-Ω resistor, and a multimeter. The area of each of the three electrodes was 0.10 m^2^. A perforated pipe (of 0.10 m length) was inserted beneath the stone media surface in the filtration chamber that was connected to an external aerator (air supply capacity of 4 L/min) through airline tubes; the purpose of external aerator integration was to supply air inside the septic tank.

The effluent produced by the septic tank was transferred to a second-stage floating wetland that had two components, i.e., a steel tank (the length, width, and height were 0.91, 0.60, and 0.46 m, respectively) and a polyvinyl chloride (PVC) - built rectangular, air buoyant frame (length: 0.74 m and width: 0.51 m); the diameter of the PVC pipe was 0.05 m. The PVC-built frame included a nylon mesh at the base to support the growth of plants. In this study, *Canna indica* were planted into the floating wetland. Such a species is often selected as a wetland plant due to its better growth and biomass production that foster microbial population development and associated metabolism (Jamwal et al., 2021). Two steel mesh-charcoal-built electrodes (i.e., one anode and one cathode) were inserted inside the water column; the area of a single electrode was 0.26 m^2^. The cathode electrode was positioned at 0.10 m depth beneath the floating mat, whereas the anode electrode was positioned at 0.10 m depth measured from the bottom of the floating tank. The electrodes in the floating tank were spaced 0.20 m apart and divided by a glass wool thermal foam. The anode and cathode electrodes of the floating wetland were connected through external electrical circuits, an 820-Ω resistor, and a multimeter.

The influent wastewater (effluent of the septic tank) was transmitted in the anode compartment of the floating wetland that passed through the hanging root network (beneath the air buoyant frame) during its passage towards the outlet located at a depth of 0.40 m (from the bottom of the tank). The floating wetland produced the final effluent of the two-stage electrode-integrated septic tank-floating wetland system. A perforated pipe (of 0.10 m length) was inserted beneath the floating mat and was connected to an external aerator (air supply capacity of 4L/min) through airline tubes for aeration purposes. After construction, the septic tank and floating wetland were waterlogged for 10 weeks to allow plant growth; previous studies recommended a growth period range of 10–12 weeks with *Canna indica* plant species in wetland systems after plantation (Saeed et al. 2022b, 2023c).

### 2.2 Sewage properties

Sewage was collected from a local outlet and stored in a feed tank. The mean environmental parameters (pH and E_h_), common pollutant concentration (nitrogen, phosphorus, organic, and solids), and coliform numbers of the sewage have been provided in Table 1. The sewage discharge standards of Bangladesh, as stated in the Environmental Conservation Rules 2023; ECR 2023), on maximum allowable environmental, common pollutant concentration, and coliform numbers, have also been provided in Table 1. The mean metals (chromium, cadmium, nickel, copper, zinc, lead, iron, and manganese) concentration profiles of the sewage are summarized in Table 2. The metals discharge guidelines of Bangladesh, as stated in the Bangladesh Standards and Testing Institution (2020); BSTI 2020), have been provided in Table 2. The BSTI (2020) standards have been formulated following the guidelines of the International Organization for Standardization (ISO 31800:2020).

**Table 1:** The mean concentration of environmental parameters and common pollutant of the collected sewage, along with sewage disposal guidelines in Bangladesh (ECR, 2023). Standard deviation values are indicated within the brackets. **The bold letters specify the mean concentration profiles of the collected sewage exceeding the discharge guidelines.**

Parameters	Unit	Concentration	ECR (2023)
pH	–	7.8 (0.5)	6–9
E_h_	mV	112.9 (71.8)	–
NH_4_–N	mg/L	43.6 (15.2)	–
NO_2_–N		0.5 (0.5)	–
NO_3_–N		10.4 (10.2)	50
TN		84.3 (18.1)	–
TP		**227.8 (168.3)**	15 (as PO_4_)
BOD		**417.1 (142.6)**	30
COD		**1552.5 (322.2)**	125
Suspended solids		**396.8 (447.9)**	100
Coliform	CFU/100 mL	**59,109 (22,147)**	1000

**Table 2:** The mean concentration of metals in the collected sewage, along with metals disposal guidelines in Bangladesh (BSTI, 2020). Standard deviation values are indicated within the brackets. **The bold letters specify the mean concentration profiles of the collected sewage exceeding the discharge guidelines.**

Parameters	Units	Concentration	BSTI (2020)
Cr	mg/L	**0.3 (0.3)**	0.1
Cd		**0.04 (0.03)**	0.01
Ni		0.2 (0.3)	0.2
Cu		0.1 (0.1)	0.2
Zn		1.2 (0.7)	2
Pb		0.1 (0.1)	5
Fe		1.4 (0.5)	5
Mn		**0.5 (0.4)**	0.2

The collected sewage was alkaline in nature, as reflected by the mean pH concentration (Table 1). The redox profiles of the sewage specify its anoxic nature. The sewage's organic biodegradation ratio (BOD/COD) was 0.3, reflecting the presence of limited biodegradable organic compounds.

### 2.3 Operational protocol

The two-stage septic tank-floating wetland was fed with the collected sewage for 52 weeks. The first 12 weeks allowed system adaptation to the composition of the sewage; an experimental campaign was conducted within the remaining 40 weeks. The collected sewage was transferred from the feeding tank to the first-stage septic tank through a timer-controlled pump at a pumping rate of 6.25 L/h. The sewage was transferred to the septic tank 16 h a day (from 8.00 a.m. to 11.59 p.m.) and was kept at resting mode (i.e., no wastewater feeding) during the remaining 8 h (12.00 p.m.-7.59 a.m.) to mimic the conditions of a real-scale septic tank. In total, 100 L of sewage was transferred to the septic tank per day with a hydraulic retention time of 24 h. The septic tank's effluent was transferred to the floating wetland through gravity drainage.

The experimental campaign was divided into four operational phases: I-IV. The duration of each phase was 10 weeks. During Phase I, the electrodes of the septic tank and the floating wetland were connected with the external resistors, referred to as the closed-circuit phase. Such closed-circuit conditions were maintained in Phases II and III. However, these phases differed in terms of external air supply rates. In Phase II, the septic tank and the floating wetland were aerated intermittently, i.e., 1 h aeration followed by 3 h non-aeration periods. The aeration and non-aeration cycles were repeated in both treatment units 24 h a day. The septic tank and the floating wetland were aerated continuously (24 h a day) in Phase III. The aerators were switched off in both treatment units during Phase IV; in addition, such units were operated as open-circuit systems (electrical circuits were not connected with external resistors). Therefore, the four phases could be defined as Phase I: non-aeration closed-circuit period; Phase II: intermittent aeration closed-circuit period; Phase III: continuous aeration closed-circuit period; and Phase IV: non-aeration open-circuit period.

### 2.4 Samples collection and analysis

The wastewater samples were collected weekly from the influent and four compartments of the septic tank throughout the four operational periods. The sampling points are specified in the schematic diagram (Fig. 1): A_0_-influent or collected sewage; A_1_-first settling chamber; A_2_-second settling chamber; A_3_-first ABR compartment; A_4_-second ABR compartment; A_5_-filtration chamber. Due to the presence of a subsequent chamber, the samples from the settling and ABR chambers (A_1_-A_4_) were collected just before reaching the corresponding outlets. Such sampling allowed discrete pollutant removal performance assessment of these compartments. On the other hand, the effluent produced by the filtration chamber was collected from the outlet (A_5_) due to the absence of a successive compartment. The effluent was also collected from the outlet (sampling point A_6_) of the floating wetland along with the septic tank samples.

The pH and E_h_ concentration of the sewage (A_0_) and wastewater samples collected from the septic tank compartments (A_1_-A_5_) and floating wetland (A_6_) were measured with an HQ 40d multi-parameter and PHC3OH, LDO101, and MTC101 probes (supplied by HACH company, USA). COD and nutrient (nitrogen compounds and phosphorus) concentrations were measured with a Kjeldahl digestion-distillation unit (supplied by VELP Scientifica, Italy), along with an Ultraviolet–visible (UV-VIS) HACH DR 6000 spectrophotometer and HACH DRB 200 reactor blocks following the protocols specified in the instrument manuals. The five-day biochemical oxygen demand (BOD_5_) concentration of the wastewater samples was quantified with HACH BOD TRAK II manometric instruments and incubators (operated at 20 °C) following the instrument manual instructions. The coliform number of the wastewater samples was quantified with Macconkey agar, and an incubator operated at a temperature of 37 °C.

The heavy metals concentration profile of the wastewater samples was measured following the standard SM3030 method. A 250 mL sample was concentrated with nitric (HNO_3_) and hydrochloric (HCl) acids for 24 h in a water bath at 80 °C; the volumetric ratio was 1 (HNO_3_): 3 (HCl). A 25 mL solution was prepared after being concentrated and filtered through a 0.45 μm filter paper. The filtrate was collected to measure the dissolved metals concentration of wastewater samples following the standard SM 3111B method and using an Atomic Absorption Spectrophotometer (model: Shimadzu AA 7000).

The media was collected from the filtration chamber of the septic tank after the termination of the experiment. Accumulated sludge was collected from the first settling chamber of the septic tank bimonthly (i.e., once every two weeks). Plants were harvested from the floating wetland (after experiment termination) and were divided into underground (UG) and aboveground (UG) portions. Common pollutant and metals accumulation in the unused (fresh), used (collected from the filtration chamber) stone media, accumulated sludge, and plant biomass was measured following the protocols described previously (Saeed et al. 2021c, 2023a).

The likely chemical composition of the unused (fresh) and used stone media (of the filtration chamber in the septic tank) was quantified energy dispersive X-ray (EDS) analysis using an energy-dispersive X-ray spectroscopy instrument (Model: EDAX Team, Manufacturer: EDAX, USA). The physical characteristics of the unused and used media were depicted through scanning electron microscopy (SEM) images that were produced with a scanning electron microscope (Model: ZEISS EVO 18, Manufacturer: Carl Zeiss Microscopy GmbH, Germany). The EDS and SEM analysis was conducted according to the protocols described in the instrument manuals (Saeed et al., 2023b).

### 2.5 Bioenergy

Voltage production across the electrode-integrated septic tank and floating wetland were measured using a connected multimeter. The current and power density production was quantified with equations (1), (2)).

(1) $$I=\frac{U}{VR}$$

(2) $$P=\frac{U^{2}}{VR}$$

Where, U = voltage production across the electrodes (millivolts, mV); R = resistance of the external resistor (Ohms, Ω); V = volume of the anode electrodes (m^3^); I = current density between the electrodes (milliamperes/m^3^, mA/m^3^); P=Power density output (milliwatts/m^3^, mW/m^3^).

## 3 Results and discussion

### 3.1 Environmental profiles

**Fig. 2:**
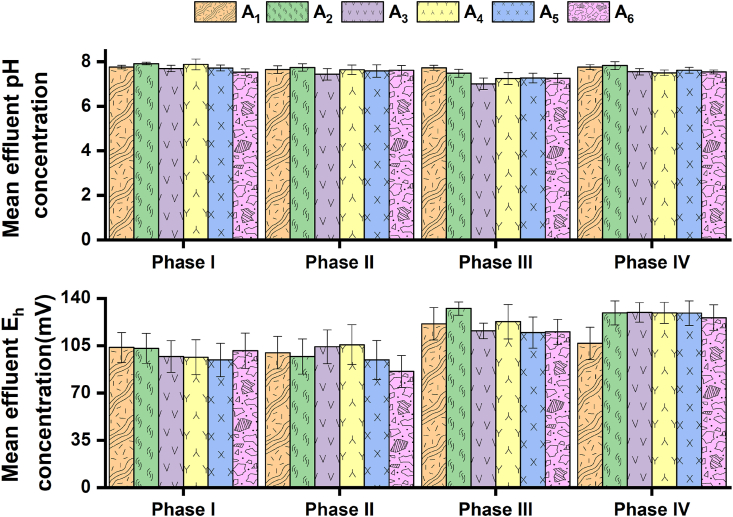
Mean effluent pH and E_h_ concentration produced in different chambers of the septic tank, along with concentration profiles of the floating wetland within the four operational phases. Bars indicate standard errors. The symbols A_1_, A_2_, A_3_, A_4_, A_5_, and A_6_ represent wastewater collection points from settling chamber 1, settling chamber 2, the first compartment of the ABR, the second compartment of the ABR, filtration chamber, and floating wetland, respectively.

Fig. 2 illustrates the mean effluent pH and E_h_ concentration produced by the four chambers of the septic tank and floating wetland within the four operational periods: I-IV. The mean effluent pH concentration ranges produced by the different components in the septic tank within the four operational periods are settling chambers: 7.5–7.9; ABR: 7–7.9; and filtration chamber/final effluent of the septic tank: 7.3–7.7. The mean effluent pH concentration produced by the floating wetland ranged between 7.3 and 7.6. Notable deviations between mean influent sewage (Table 1) and effluent pH concentration profiles (Fig. 2) were not observed. Therefore, the operational protocol variations (in terms of aeration and circuit arrangements) or structural variations of the compartments did not influence the effluent pH profiles of the septic tank and floating wetland.

The mean effluent redox profile ranges across the four chambers of the septic tank (within the four operational periods) are settling chambers: 97–133 mV; ABR: 96–130 mV; and filtration chamber/final effluent of the septic tank: 94–129 mV. The mean effluent E_h_ profiles ranged between 85 and 126 mV in the floating wetland.

### 3.2 Common pollutant removal: organic compounds and nutrient

#### 3.2.1 Physicochemical removal in settling chambers and ABR of the septic tank

**Fig. 3:**
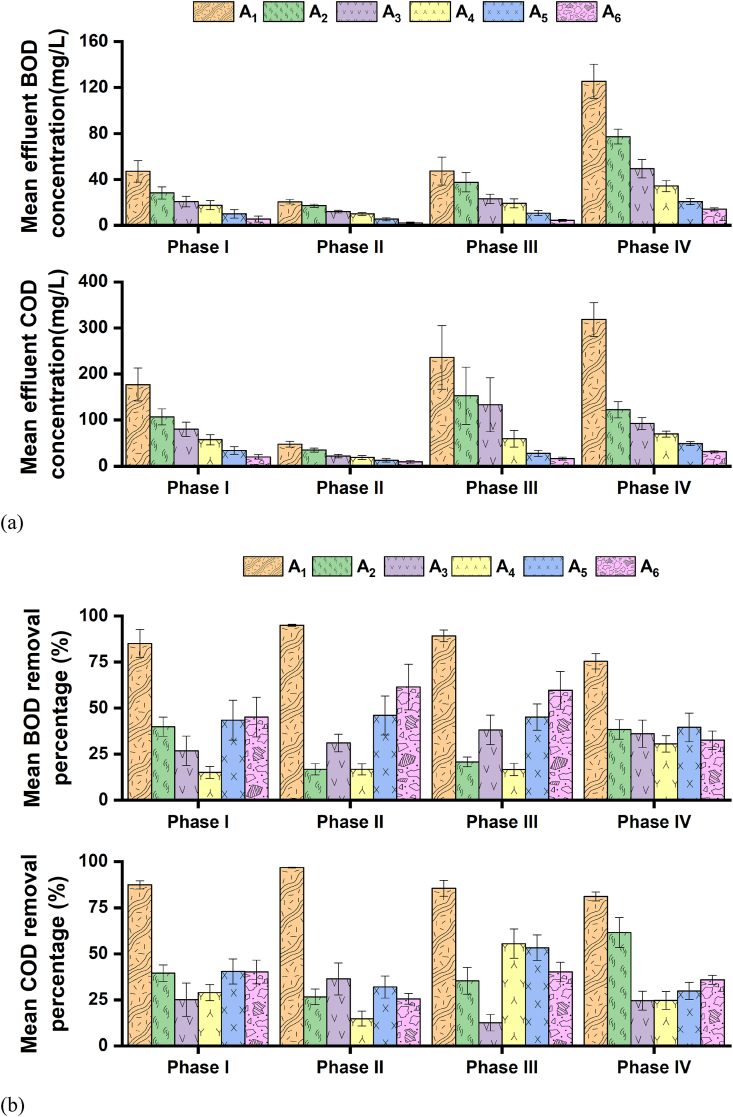
Organic removal profiles: (a) mean effluent BOD and COD concentration produced in different chambers of the septic tank, along with concentration profiles of the floating wetland within the four operational phases; and (b) associated removal percentages. Bars indicate standard errors. The symbols A_1_, A_2_, A_3_, A_4_, A_5_, and A_6_ represent wastewater collection points from settling chamber 1, settling chamber 2, the first compartment of the ABR, the second compartment of the ABR, filtration chamber, and floating wetland, respectively.

**Fig. 4:**
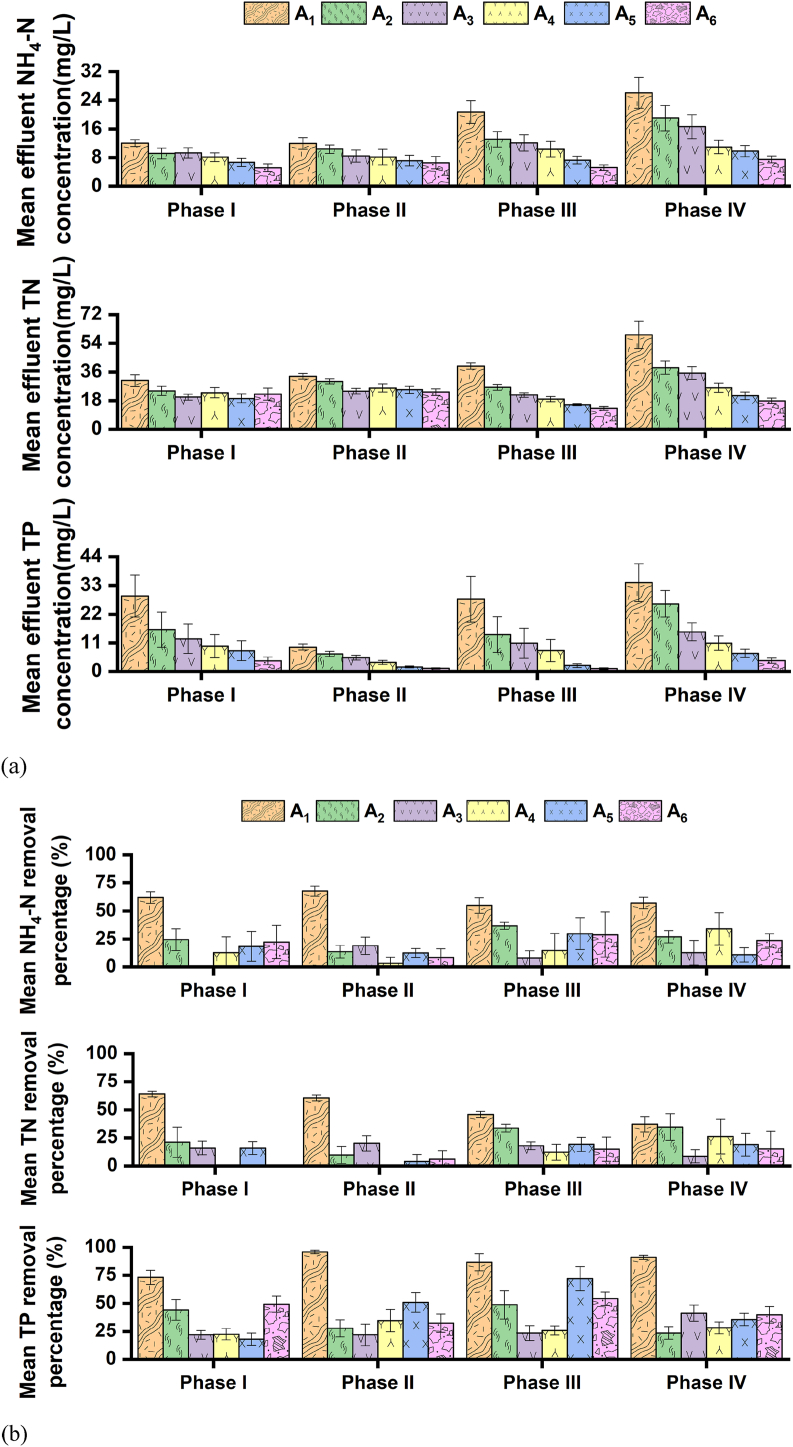
Nutrient removal profiles: (a) mean effluent NH_4_–N, TN, and TP concentration produced in different chambers of the septic tank, along with concentration profiles of the floating wetland within the four operational phases; and (b) associated removal percentages. Bars indicate standard errors. The symbols A_1_, A_2_, A_3_, A_4_, A_5_, and A_6_ represent wastewater collection points from settling chamber 1, settling chamber 2, the first compartment of the ABR, the second compartment of the ABR, filtration chamber, and floating wetland, respectively.

Fig. 3, Fig. 4 illustrate the mean effluent organic (BOD and COD) and nutrient (nitrogen and phosphorus) concentration profiles and removal percentages of the settling chambers and the ABR of the septic tank within the four operational periods: I–1V. The two settling chambers of the septic tank achieved a mean 90–98% COD, 84–96% BOD, 68–73% NH_4_–N, and 58–72% TN removal percentage ranges throughout the four operational periods. Influent organic matter and nitrogen are often removed by physicochemical pathways (i.e., settling, entrapment, and adsorption) induced by the accumulated sludge in the settling chambers of septic tanks (Hu et al., 2020; Nasr and Mikhaeil, 2013; Saeed et al., 2021a; Sharma et al., 2014). Therefore, the chemical composition of the sludge that accumulated in the first settling chamber (of the septic tank in this study) was quantified (section 2.4); Table 3 summarizes the chemical composition of the accumulated sludge. As observed in Table 3, the COD, NH_4_–N, NO_3_–N, and TN concentration of the sludge (that accumulated in the first settling chamber) was substantially higher than the sewage profiles (Table 1). These trends suggest the contribution of settling, entrapment, and assimilation pathways, resulting in observed concentration increment of the accumulated sludge in the settling chambers.

**Table 3:** The mean composition of the sludge accumulated in the first settling chamber. Standard deviation values are presented within the brackets.

	Parameters	Units	Concentration
Common pollutant	pH	–	8.0 (0.6)
	COD		4326.0 (934.9)
	NH_4_–N	mg/L	115.8 (79.8)
	NO_2_–N		2.4 (1.6)
	NO_3_–N		478.8 (455.3)
	TN		727.1 (523.5)
	TP		1307.6 (553.7)
	TSS		20,293 (9633.0)
	Coliform	CFU/100 mL	45,980.0 (16,330.0)
Metals	Cr	mg/L	0.7 (0.4)
	Cd		0.4 (0.2)
	Ni		1 (0.3)
	Cu		4.8 (1.5)
	Zn		170.5 (32.2)
	Pb		1.4 (0.3)
	Fe		17.6 (1.7)
	Mn		13.2 (3.6)

The subsequent ABR achieved mean BOD and COD removal percentages of 37–56% and 43–62%, respectively, throughout the four operational periods. The ABR also achieved notable overall mean NO_3_–N removal percentages, i.e., 48–76% throughout the experimental campaign. The mean organic (BOD and COD) and NO_3_–N concentrations in the wastewater decreased as it passed from the first to the second compartment of the ABR. Hence, physical separation and assimilation induced by the settled biomass probably contributed to organic and nitrogen removal as the wastewater was forced to pass (by the baffle wall) from the first to the second ABR compartment (Nasr and Mikhaeil, 2013).

The settling chambers and the ABR achieved a mean of 85–97% and 39–58% TP removals, respectively. Higher phosphorus concentration of the accumulated sludge compared to sewage profiles (Table 1, Table 3) fortify a major contribution by adsorption pathways in removing influent phosphorus. Chemical-based wastewater phosphorus adsorption is primarily induced by the presence of Fe/Ca/Al elements (Mlih et al., 2020; Vohla et al., 2011). Substantial Fe concentration was observed in accumulated sludge (Table 3), which could have favored phosphorus adsorption.

#### 3.2.2 Adsorption and microbial decomposition in the filtration chamber of the septic tank

The organic and nutrient removal profiles in the filtration chamber of the septic tank have been provided in Fig. 3, Fig. 4. The mean COD and BOD removal percentage ranges in the filtration chamber (of the septic tank) were 39–46% and 30–54%, respectively, within the four operational periods. The biodegradable and non-biodegradable organic compounds that escaped the treatment mechanisms of the preceding settling chambers and ABR were probably trapped by the media pores or decomposed by the attached biofilm in the filtration chamber (GarcÍA et al., 2010; Han et al., 2019; Saeed et al., 2021a).

The filtration chamber achieved mean NH_4_–N and TN removal percentage ranges (within the four operational periods) of 10–30% and 4–20%, respectively. Nitrogen removal in the filtration chamber was probably supported by two distinct pathways: media-based chemical adsorption and microbial decomposition. The adsorption pathway is activated by the presence of potassium (K^+^), sodium (Na^+^), calcium (Ca^2+^), and magnesium (Mg^2+^) cations in the media; these cations form ion exchange sites on the media surface to promote wastewater NH_4_–N adsorption (Damodara Kannan and Parameswaran, 2021; Xie et al., 2020). To assess the probable existence of adsorption-inducing cations, EDS analysis of the unused (fresh) and used (collected from the filtration chamber after experiment termination) stone media was conducted (section 2.4). The probable elemental composition of such materials is presented in Supplementary material S1. EDS analysis signifies the probable presence of the specific cations in both unused (fresh) and used stone media (of the filtration chamber), suggesting the existence of NH_4_–N adsorption that contributed to observed nitrogen removal (Fig. 4) in the filtration chamber. Concentration profiles analysis of the unused (fresh) and used stone media (of the filtration chamber) specify higher nitrogen concentration in the used media compared to unused media composition (Table 4). As such, the results of EDS (Supplementary material S1) and media concentration analysis (Table 4) confirm the contribution of media-based NH_4_–N adsorption on observed nitrogen removal in the filtration chamber.

**Table 4:** Pollutant concentration analysis of the unused and used stone media in the filtration chamber.

Parameters	Unused (mg/kg)	Used (mg/kg)
N	1000	1600
P	600	1200
Cr	255.4	4.4
Cd	14.7	0.3
Ni	11.2	5.9
Cu	22.2	3.1
Zn	28.6	1455.0
Pb	12.9	0.8
Fe	810.3	1944.7
Mn	86.5	31.9

The mean NO_3_–N removals ranged between 20 and 30% in the filtration chamber, reflecting the coexistence of classical denitrification (along with adsorption pathways), i.e., NO_3_–N reduction to N_2_ gas. In a media-based and electrode-integrated wastewater treatment system, NO_3_–N contents are microbially decomposed by the attached biofilms (Ren et al., 2021). The SEM images of the unused and used media (section 2.4) depict the smooth surface structure of the unused stone materials (Supplementary material S2); the used stone media (of the filtration chamber) was covered with aggregate-like depositions that the biofilms could have contributed. Microbial denitrification (along with media-based chemical adsorption) could have existed in the deeper layer of such formed biolayer (Doherty et al., 2015).

The filtration chamber of the septic tank achieved a mean 36–73% phosphorus removal percentage range throughout the experimental campaign. EDS analysis suggests the probable presence of phosphorus-binding elements (Fe, Al, and Ca) in the unused and used stone media (Supplementary material S1). Such composition could have supported additional phosphorus removal in the filtration chamber and is also reflected by the phosphorus concentration increment of the used stone media compared to unused (fresh) media profiles (Table 4).

#### 3.2.3 Hanging plant roots-based pollutant removal in the floating wetland

The organic and nutrient removal profiles in the second stage floating wetland have been provided in Fig. 3, Fig. 4. The second stage wetland achieved a mean COD and BOD removal percentages of 25–41% and 32–62%, respectively, within the four operational periods. The maximum mean NH_4_–N and TN removal percentages of the floating wetland were 29% and 16%, respectively. The floating wetland achieved overall mean TP removal percentages ranging between 32 and 55% throughout the four operational periods. These overall removals indicate that the second-stage floating wetland further reduced the effluent organic and nutrient concentration of the first-stage septic tank.

Nutrient removal in floating wetland is primarily achieved through two pathways: (a) uptake by the hanging plant root mass; and (b) microbial decomposition in the bulk rootzone (Barco and Borin, 2020; Headley and Tanner, 2012). The probable contribution of the first pathway is assessed through Table 5, which summarizes the nitrogen concentration of AG and UG biomass of the *Canna indica* plant that was integrated with the floating wetland of this study. According to the data sets of Table 5, nitrogen accumulation in both segregated plant portions was observed, reflecting the existence of uptake kinetics. Phosphorus removal also followed similar trend, i.e., phosphorus accumulation in AG and UG portions of *Canna indica* (Table 5) planted in the floating wetland. The probable influence of the second pathway (i.e., microbial decomposition) on nutrient removal will be further discussed in section 3.4.

**Table 5:** Nutrient and metals concentration in aboveground (AG) and underground (UG) biomass of *Canna indica*.

Parameters	Plant portions	Concentration (mg/kg)
N	AG	12,000
	UG	15,000
P	AG	1200
	UG	2100
Cr	AG	1.9
	UG	4.7
Cd	AG	3.5
	UG	6.8
Ni	AG	4.2
	UG	13.5
Cu	AG	12.4
	UG	14.3
Zn	AG	1562.0
	UG	4952.0
Pb	AG	26.1
	UG	48.5
Fe	AG	1061.9
	UG	2267.6
Mn	AG	251.8
	UG	834.2

### 3.3 Metals removal

#### 3.3.1 Overall removal performances

**Fig. 5:**
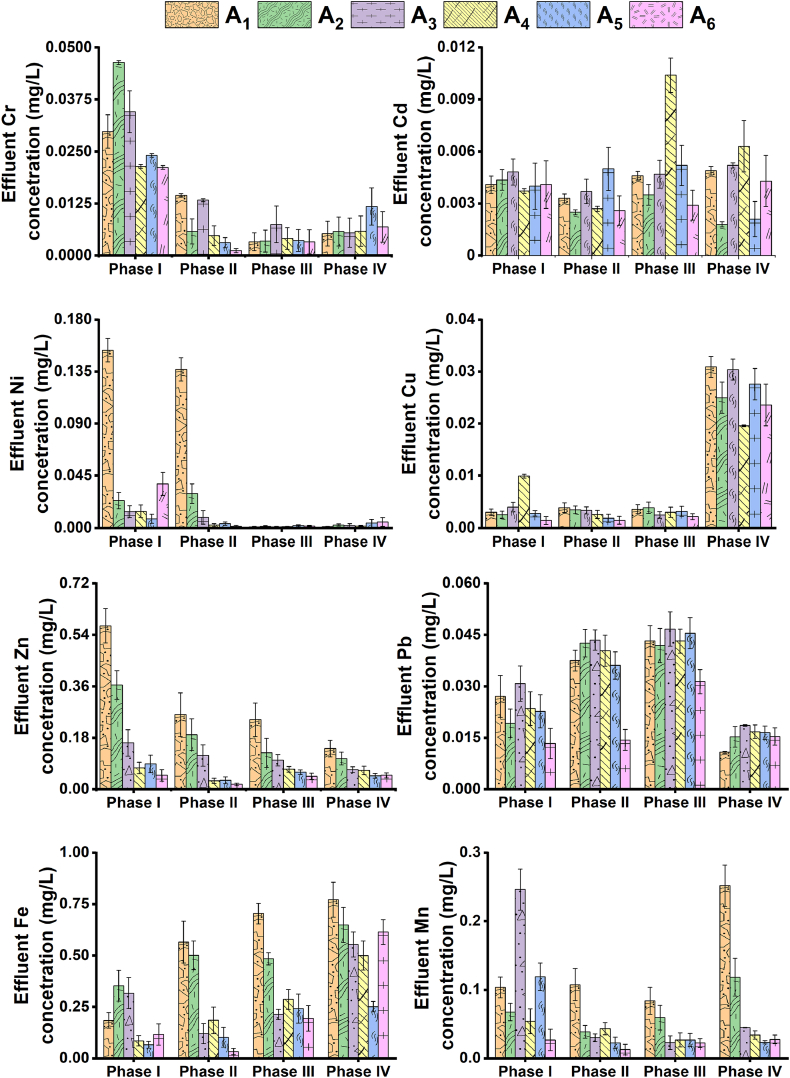
The mean effluent metals (Cr, Cd, Ni, Cu, Zn, Pb, Fe, and Mn) concentration produced in different chambers of the septic tank, along with concentration profiles of the floating wetland within the four operational phases. Bars indicate standard errors. The symbols A_1_, A_2_, A_3_, A_4_, A_5_, and A_6_ represent wastewater collection points from settling chamber 1, settling chamber 2, the first compartment of the ABR, the second compartment of the ABR, filtration chamber, and floating wetland, respectively.

Fig. 5 illustrates the mean effluent metals (Cr, Cd, Ni, Cu, Zn, Pb, Fe, and Mn) concentration profiles of the four chambers in the septic tank and the overall effluent concentration of the second-stage floating wetland within the four operational periods: I–1V. The mean effluent Cr, Cd, Ni, Cu, and Pb concentration ranged between 0.001 and 0.2 mg/L within the two settling, ABR and filtration chambers. The mean effluent Zn, Fe, and Mn concentration ranges were marginally higher, i.e., between 0.02 and 0.8 mg/L, probably due to their higher mean concentration values and greater variations (indicated by higher standard deviation values) in the influent (Table 2). The first settling chamber achieved a mean 51–98% metals removal (within the four operational periods) that exceeded the removals of the subsequent second settling, ABR, and filtration chambers in the septic tank. Such following chambers achieved a more dispersed metals removal percentage range of 10–90%, along with irregular negative removals, i.e., effluent metals concentration increments over influent profiles. Mean effluent metals concentration across the second-stage floating wetland was very low, ranging between 0.003 and 0.2 mg/L; mean removal percentages ranged between 81 and 98%, indicating additional metals removal from the effluent of the first-stage system.

#### 3.3.2 Precipitation and co-precipitation

Wastewater metals removal is often achieved through abiotic pathways such as precipitation and co-precipitation (Batool and Saleh, 2020; Bavandpour et al., 2018). An alkaline environment favors metals removal because of the forming of Cu, Zn, Pb, and Cd hydroxides and subsequent precipitation (Yu et al., 2022). The influent sewage (across the septic tank) and the accumulated sludge were alkaline in nature, as signified by the mean pH concentration profiles (Table 1, Table 3). Metals concentration increase in the accumulated sludge (Table 3) compared to sewage composition (Table 2), along with Fe and Zn concentration increment of the used media in the filtration chamber compared to the unused media profiles (Table 4), designate wastewater alkalinity-driven metals precipitation. Metals could also co-precipitate with Fe and Mn oxyhydroxides (Yu et al., 2022); a sharp Fe and Mn concentration increment was observed in the accumulated sludge of the first settling chamber (Table 3), signifying the probable existence of metals co-precipitation.

#### 3.3.3 Metals adsorption and plant-based accumulation

Apart from precipitation and co-precipitation, wastewater metals, particularly Cr could also be removed by adsorption induced by the precipitated Fe and Mn complexes (Chen et al., 2021; Sultana et al., 2014). Such a chemical pathway might have allowed Cr retention in the accumulated sludge due to its Fe and Mn concentration increment (Table 3). A few studies reported the positive impact of organic matter on metals removal due to reactivity between metals and organic components such as humic compounds, chitin, and lignin (Chen et al., 2021; Saeed et al., 2021b). The substantial COD composition of the accumulated sludge (in the first settling chamber-Table 3) could have supported such bondage formation between metals and organic compounds (of the accumulated sludge), thus allowing metals retention.

Unstable metals removal performance of the second settling chamber, ABR, and filtration chamber might be attributed to precipitated Fe/Mn flocs disintegration and deposited organic matter degradation that might have disrupted the bondage formed between metals and Fe/Mn flocs or organic matter and allowed metals leaching into bulk wastewater (Saeed et al., 2023a). Regardless of such unstable metals removals in second settling, ABR, and filtration chambers, the septic tank produced very low mean effluent metals concentration (except Fe) ranging between 0.002 and 0.06 mg/L throughout the experimental campaign, thus demonstrating its metals removal efficacy. Probable disintegration of the deposited Fe flocs could have produced slightly higher mean effluent Fe concentration ranges, i.e., 0.07–0.3 mg/L, across the septic tank. Metals concentration decrease (except Zn and Fe) with the used stone media (of the filtration chamber) compared to the unused media profiles (Table 4) was an interesting pattern observed in this study. These trends indicate the probable existence of a route that caused metals loss from the filler media of the septic tank. A probable explanation of such disappearance could be attributed to the metals binding affinity with the organic components (Saeed et al., 2023a). The organic constituents of the aggregate-like depositions of the used media (Supplementary material S2) might have favored such bondage formation, resulting in metals concentration decrease in the used stone media. However, this hypothesis could not be proved in this study and should be focused in future studies. With the second-stage floating wetland, metals accumulation was observed in the UG and AG portions of *Canna indica* plants (Table 5), signifying the influence of biotic pathways (Barco and Borin, 2020).

### 3.4 Impact of operational variables: circuit connection and aeration

The previous sections (3.2 and 3.3) provide an in-depth discussion on pollutant removal pathways and pollutant partitioning among the septic tank and floating wetland components, which was an objective of this study. This section discusses the impact of operational variables, i.e., circuit connection and external aeration, on pollutant removal performance of the developed two-stage and electrode-integrated septic tank-floating wetland system, another objective of this study.

#### 3.4.1 Circuit connection

The organic removal performance comparison of the ABR (where the anode plates were located) in the septic tank between non-aeration dependent closed (Phase I) and open circuit (Phase IV) periods indicates better COD removal in Phase I (i.e., 47%) compared to the removal (i.e., 43%) of the same reactor in Phase IV. Electrochemically active and inactive removal pathways coexist with bioenergy-producing wastewater treatment systems (Gupta et al., 2021). Such coexistence might have improved the organic removal performance of the ABR during closed circuit protocol, a unique advantage of the electrochemical systems that was also reported previously (Saeed et al., 2022b). The open circuit protocol could have disrupted such electrochemically active organic matter oxidation (Srivastava et al., 2020), resulting in a major involvement of the electrochemically inactive pathway in removing organic matter. The contribution from a single removal route could have diminished the ABR chamber's organic removal that decreased overall organic removal performance of the septic tank in Phase IV compared to Phase I performance.

Electrochemical-based bioreactions could also assist in removing wastewater nitrogen, as reported in previous studies (Srivastava et al., 2020; Tang et al., 2019). In this study, the mean TN removal of the ABR was lower, i.e., 5% during the non-aeration closed circuit protocol (Phase I) compared to 33% mean TN removal (of the same reactor) during the non-aeration open-circuit period (Phase IV). Therefore, it could be stated that nitrogen removal in the ABR was not dependent on electrochemical-based bioreactions and was likely to be influenced by the establishment of a matured biofilm inside the ABR as the experiment advanced. The mean effluent phosphorus concentration values across the septic tank did not deviate notably within the non-aeration dependent closed (Phase I) and open-circuit (Phase IV) protocols and ranged between 7 and 8 mg/L. These trends suggest that phosphorus removal did not rely upon the existence of electrochemical-based reactions in the septic tank.

Regarding the impact of circuit connection mode on the second-stage floating wetland, the system produced lower effluent mean effluent organic concentration during non-aeration closed circuit Phase I (BOD: 6 mg/L; COD: 21 mg/L) compared to the non-aeration open-circuit Phase IV (BOD: 14 mg/L; COD: 32 mg/L). Such a trend was similar to the septic tank, reflecting the additional contribution of electrochemical-based organic matter oxidation to improve organic removal. Mean effluent TN concentration across the floating wetland was higher, i.e., 22.1 mg/L during the non-aeration closed circuit period (Phase I), than the performance, i.e., 17.9 mg/L of the non-aeration open-circuit protocol (Phase IV). A similar trend was observed with the septic tank that was attributed to the probable establishment of a matured biofilm. Microbial-based nitrogen removal via denitrification has been reported as the major nitrogen removal mechanism with floating wetlands (Barco and Borin, 2020).

#### 3.4.2 External aeration

The intermittent and continuous aeration protocols produced lower overall mean effluent COD (12–28 mg/L) and BOD (5–11 mg/L) concentration across the septic tank compared to non-aeration periods performance (COD: 33–49 mg/L; BOD: 10–21 mg/L). Mean COD and BOD removal percentage ranges of 85–97% and 89–95%, respectively, were observed in the first settling chamber during aeration periods that exceeded its non-aeration periods-based performances, i.e., 81–88% COD and 75–86% BOD removals. These trends suggest that the external air supply could have triggered electrochemically inactive aerobic microbial decomposition in the sludge layer of the settling chambers (Teoh et al., 2020). Moreover, the ABR of the septic tank achieved a mean of 45–62% COD removal ranges during closed-circuit dependent intermittent and continuous aeration periods II and III, respectively; these performances exceeded its corresponding 43–47% mean COD removal ranges within non-aeration periods I and IV. The external air supply in the cathode compartment increases oxygen availability and assists in the completion of electrochemical bioreactions (Oon et al., 2017; Saeed et al., 2023b; Srivastava et al., 2017). As such, improved organic removals of the settling chambers and the ABR contributed to overall organic removals improvement of the septic tank during aeration periods.

Regarding the influence of air supply rate differences (i.e., intermittent vs. continuous aeration) on closed-circuit operational protocols, the ABR (of the septic tank) achieved a mean of 62% COD and 49% BOD removals during Phase III (continuous aeration, closed circuit). Such performances exceeded its corresponding mean COD (46%) and BOD (43%) removals of Phase II (intermittent aeration, closed circuit). Continuous air supply could have increased oxygen availability around the cathode electrode (for completing electrochemical oxidation) in the filtration chamber of the septic tank that received anoxic influent. It should be noted that although continuous aeration improved the organic removal performance of the ABR component, the mean effluent BOD (5.4 mg/L) and COD (12.7 mg/L) concentration of the septic tank was lower during the intermittent aeration period compared to that (BOD: 10.6 mg/L; COD: 27.6 mg/L) of the continuous aeration period. A mean of 5–12% decrease in the removal of organic matter was observed in the settling chambers during the continuous aeration period compared to the removals of the intermittent aeration phase. Continuous air supply increased turbulence in the bulk water that might have resuspended a portion of the settled particulates from the accumulated sludge, thus undermining the improved organic removals of the ABR and resulting in an overall organic removal performance decrease of the septic tank (compared to intermittent aeration-based performance).

The ABR achieved a lower mean effluent TN concentration (19 mg/L) during the continuous aeration period compared to its mean effluent TN concentration ranges of 23–27 mg/L produced during the other three phases. The overall performance of the septic also exhibited a similar trend: lower mean effluent TN concentration production (15.3 mg/L) during the continuous aeration period than the other three periods (19–25 mg/L). These results suggest that constant air supply positively influenced nitrogen transformation routes, i.e., ammonification and nitrification, due to their dependency on oxygen availability (Oon et al., 2017; Saeed et al., 2021c).

The mean effluent phosphorus concentration ranges of non-aeration periods (Phases I and IV) were substantially high (7–8 mg/L) when compared to mean effluent phosphorus concentration profile ranges, i.e., 1.7–2.3 mg/L, produced by the septic tank during closed-circuit dependent intermittent and continuous aeration periods (Phases II and III). Better phosphorus removal performance of the septic tank during aeration periods could be linked to the dependency of Fe adsorption sites on surrounding redox potentials; low redox gradient causes the release of Fe-bound phosphorus and vice versa at high potentials (Mlih et al., 2020). Minor mean effluent phosphorus concentration deviation across the septic tank within intermittent and continuous aeration periods signifies that the air supply rate differences did not influence overall phosphorus removal.

The mean effluent metals concentration profiles produced by the septic tank within non-aeration-dependent closed and open-circuit periods did not exhibit any specific trend. On the other hand, the septic tank achieved the lowest mean effluent metals concentration ranges, i.e., between 0.002 and 0.1 mg/L during intermittent and continuous aeration periods. Aerobic environments create favorable conditions to form Fe and Mn oxyhydroxides and their co-precipitation with other metals (Yu et al., 2022). This is also supported by the production of mean effluent Fe concentration ranges, i.e., 0.03–0.2 mg/L during aeration periods that were beneath the profiles, i.e., 0.1–0.6 mg/L of the non-aeration periods. However, the positive influence of continuous over intermittent aeration on metals removal in the septic tank could not be confirmed in this study due to minor effluent concentration differences between the two operational protocols.

The organic removal with the second-stage floating wetland improved because of intermittent and continuous aeration (effluent concentration BOD: 2–5 mg/L; COD: 9–17 mg/L), compared to the non-aeration periods (effluent concentration BOD: 5–14 mg/L; COD: 20–32 mg/L). The former approach was more efficient in improving organic removals, which was contradictory to the ABR (of the septic tank) performance but coincided with the overall performance of the septic tank. It should be noted that plants contribute to oxygen supply through root-based leakage in constructed wetlands (Barco and Borin, 2020). Therefore, intermittent aeration appeared to be a more effective approach due to: (a) matching the oxygen supply by plants for maintaining the required redox gradient between the electrodes to complete electrochemical organic matter oxidation; and (b) minimizing probable turbulence in the bulk water and associated resuspension of the trapped particulates from the bulk hanging root. The floating wetland achieved the lowest mean effluent TN concentration (13 mg/L) during the continuous aeration period compared to those (17–24 mg/L) of the other three phases; air availability could have supported aerobic-based nitrogen transformation routes (Teoh et al., 2020) that produced more NO_3_–N for denitrification. The floating wetland produced lower mean effluent phosphorus concentration ranges, i.e., 1–1.5 mg/L during intermittent and continuous aeration periods (Phases II and III) compared to non-aeration periods (Phases I and IV), i.e., 4–4.5 mg/L. Therefore, external air availability positively influenced phosphorus removal performances of the floating wetland, probably due to the presence of Fe in the UG plant biomass portion (Table 5) and its dependency on the redox gradient to bound phosphorus (Mlih et al., 2020).

Although metals accumulation was observed in the UG and AG portions of *Canna indica* plants (Table 5), signifying the influence of biotic pathways, very low effluent metals (except Fe) concentration ranges, i.e., 0.001–0.01 mg/L during aeration periods, also demonstrate the positive influence of external air availability on the associated abiotic metals removal pathways with the floating wetland. In general, the overall trends demonstrate the coexistence of biotic and abiotic pathways in the floating wetlands that allowed additional metals to be removed from the septic tank effluent. The removals were further improved by intermittent or continuous aeration but independent of closed or open-circuit protocols.

### 3.5 Coliform removal of the two-stage system

The effluent quality produced from onsite or decentralized wastewater treatment systems is often expected to meet reuse guidelines; coliform numbers in the effluent are a major parameter for reuse, particularly for agricultural purposes (Sánchez et al., 2023; Tripathi et al., 2019). Therefore, coliform numbers of the effluent produced from the two-stage system of this study were assessed. The mean effluent coliform numbers and removal percentages of the four chambers in the septic tank and the overall performance of the second-stage floating wetland within the four operational periods have been provided in Supplementary material S3.

The septic tank achieved a mean coliform removal of 92% throughout the experimental run. Wastewater coliform removal is primarily achieved through physical (sedimentation, filtration, and UV radiation), chemical (oxidation, adsorption, and microbial, plant-based toxin production), and biological (predation and natural decay) pathways (Barco and Borin, 2020; Colares et al., 2021). Coliform presence was observed in the accumulated sludge of the first settling chamber (Table 3); hence, physical processes, i.e., sedimentation and entrapment of coliform-bound particulates by the accumulated sludge, might have contributed to coliform separation from wastewater (Saeed et al., 2023a). Coliform removal was also observed in the filtration chamber (Supplementary material S3), probably influenced by media-oriented physical processes, i.e., sedimentation and filtration (Barco and Borin, 2020). The septic tank produced the lowest mean effluent coliform numbers (i.e., 3670 CFU/100 mL) during the intermittent aeration closed circuit period (Phase II), followed by 3918 CFU/100 mL during the non-aeration closed circuit protocol (Phase I). Current generation and oxygen-rich environment increase wastewater coliform mortality (Barco and Borin, 2020; Colares et al., 2021; Saeed et al. 2023b, 2023c). However, the septic tank produced a mean effluent coliform number of 4650 CFU/100 mL during the continuous aeration closed circuit period (Phase III). These profiles exceeded the effluent coliform numbers of the previous two operational periods. Bulk water turbulence caused by continuous aeration might have triggered resuspension of the settled coliform from the accumulated sludge and filler media pores, resulting in an increment in effluent coliform numbers.

The floating wetland achieved a mean of 59% coliform removal percentages throughout the experimental campaign. The hanging plant roots of the floating wetland might have assisted in removing influent coliform through filtration, sedimentation, and adsorption of the coliform-bound particulates (Ansa et al., 2012; Colares et al., 2021). The floating wetland achieved the lowest mean effluent coliform numbers of 1410 CFU/100 mL during the continuous aeration closed circuit phase, followed by 1681 CFU/100 mL in the non-aeration dependent closed-circuit protocol. Such a performance suggests a synergetic positive impact of constant air supply and current flow on coliform mortality that was observed with the septic tank in the case of intermittent aeration protocol. The presence of hanging roots inside the water column of the floating wetland might have diminished water turbulence (induced by constant air supply) and associated coliform resuspension.

### 3.6 Bioenergy production

#### 3.6.1 Voltage and power density

**Fig. 6:**
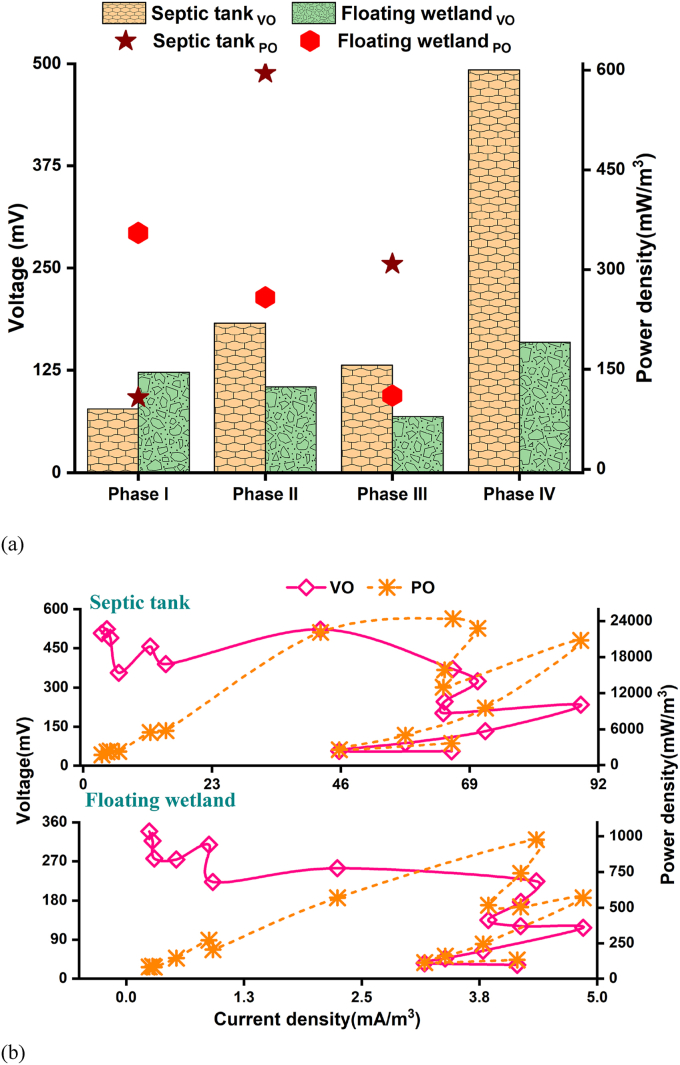
Bioenergy profiles: (a) mean voltage and power density production of the septic tank and the floating wetland during the four operational periods; and (b) polarization curves.

The bioenergy production capacity of the electrochemical-based wastewater treatment technologies often indicates their overall operational efficiencies (Gupta et al., 2021). Therefore, a bioenergy production assessment of the developed electrode-integrated septic tank and floating wetland was conducted, which was a secondary objective of the study. Fig. 6a represents the mean voltage production of the septic tank and floating wetland within the four operational periods (I-IV) and their power density production performance during the first three phases (i.e., closed circuit protocols: I-III). The septic tank produced mean voltage of 78, 183, and 132 mV during closed-circuit dependent non-aeration (Phase I), intermittent (Phase II), and continuous aeration (Phase III) periods, respectively. The mean power density production of the septic tank was 108, 596, and 309 mW/m^3^ during Phases I, II, and III, respectively. These trends signify better bioenergy production performance of the septic tank during aeration periods (Phase II and III) compared to non-aeration phase (I)-based performance. Limited oxygen availability in the cathode compartment and bioenergy production decrease of the electrochemical technologies are positively correlated; such an operational disadvantage could be counterbalanced by external air supply in the cathode compartment (Guadarrama-Pérez et al., 2019; Oon et al., 2017). During intermittent aeration, the septic tank achieved higher power output than continuous aeration-based performance. Two hypotheses could be proposed for such performance deviation because of the difference in air supply rates. First, a constant air supply could have suppressed the anoxic/anaerobic zone in the septic tank required for electrochemical organic matter oxidation and associated electron production (Srivastava et al., 2017). Second, continuous air supply could have triggered electron (produced from electrochemical organic matter oxidation pathway) consumption by electrochemically inactive microbial population (before being captured by the anode electrode), which is often referred to as electron losses (Saeed et al., 2023b). The septic tank produced higher voltage during open circuit protocol (Phase IV) than the preceding closed-circuit operational phases I-III. Higher voltage production of the open circuit system was also reported previously, which was linked to the absence of two factors: internal ohmic resistance development and over-potential of the electrode (Srivastava et al., 2020).

The floating wetland achieved mean voltage and power density production ranges of 68–123 mV and 110–355 mW/m^3^, respectively, within the closed-circuit phases (I-III). A higher mean voltage production of 160 mV was observed during the open circuit protocol, a phenomenon that coincided with the septic tank. The bioenergy production deviation of the floating wetland because of the difference in air supply rates contradicted the septic tank: better production during the non-aeration phase (I) compared to the aeration periods (II and III). Moreover, the septic tank was more efficient in bioenergy production than the floating wetland. Electrochemical organic matter oxidation predominantly depends upon the biodegradable organic matter composition of wastewater (Gupta et al., 2021). The organic matter removal in the preceding septic tank and its more efficient performance during aeration periods diminished biodegradable organic matter proportions in the influent of the floating wetland. Such limited biodegradable organic matter availability might have adversely influenced electrochemical organic matter oxidation in the second-stage floating wetland.

#### 3.6.2 Polarization test

The polarization test provides an insight into probable internal resistance development between the electrodes of bioenergy-producing systems; the power output efficiencies of such technologies are negatively correlated with internal resistance development (Saeed et al., 2022b; Srivastava et al., 2020). In this study, external resistors ranging between 100 and 33,000 Ω were connected with the septic tank and the floating wetland for conducting polarization tests and assessing internal resistance. The current, power density, and voltage produced due to connecting variable external resistors have been plotted in Fig. 6b, which is commonly referred to as the polarization curve. As observed in Fig. 6b, maximum power output of 24,413 and 977 mW/m^3^ was observed with the septic tank and the floating wetland, respectively, when connected to an external resistor of 1000 Ω. Therefore, internal resistance developed in the septic tank and floating wetland during closed circuit protocols that restricted its voltage production capacity compared to open circuit phase-based performance.

### 3.7 Research implication

The septic tank achieved better common pollutant (organic, nutrient, and coliform) and metals (Cr, Cd, Ni, Cu, Zn, Pb, Fe, and Mn) removal due to the coexistence of physicochemical and microbial pathways in the four chambers. The integration of a floating wetland further polished the effluent of the septic tank (as observed in this study). The overall mean effluent BOD, COD, TN, TP, and suspended solids concentration produced by the two-stage septic tank-floating wetland (of this study) was 7, 20, 19, 3, and 2 mg/L, respectively, that was beneath the ECR 2023 guidelines (Table 1) for sewage disposal in Bangladesh. Moreover, such profiles might allow for achieving a primary objective of the decentralized wastewater systems, i.e., effluent reuse. However, the mean effluent coliform number (i.e., 2047 CFU/100 mL) produced by the two-stage septic tank-floating wetland (in this study) might not fulfill stringent reuse guidelines. Therefore, future studies should be undertaken focusing on circuit connections and aeration strategies to reduce effluent coliform concentration that could fulfill stringent effluent reuse criteria. The two-stage system of this study achieved mean organic, nitrogen, phosphorus, and coliform removal percentages of 99, 78, 99, and 97%, respectively, primarily because of greater removal performances of the first-stage septic tank, followed by additional removal in the second-stage floating wetland (Fig. 3, Fig. 4 and Supplementary material S3), resulting in synergetic enhanced removal performances. These removal percentages exceeded the organic (89%), nitrogen (61%), and phosphorus (67%) removals of the integrated septic tank-constructed wetland systems employed worldwide for rural wastewater treatment (Moreira and Dias, 2020).

The Cr, Cd, and Mn concentrations in the sewage exceeded the corresponding discharge guidelines in Bangladesh (Table 2). The overall mean effluent Cr, Cd, and Mn concentration of the two-stage system in this study was 0.008, 0.003, and 0.02 mg/L, respectively, which was substantially beneath the respective discharge standards in Bangladesh (Table 2). The mean effluent Ni, Cu, Zn, Pb, and Fe concentration ranges of the two-stage system were 0.007–0.2 mg/L and were beneath the corresponding concentration values of the sewage (Table 2). It should be noted that although concentration profiles of such metals in the sewage fulfilled the disposal guidelines (Table 2), their overall removal percentages (81–97%) and associated kinetics, as observed in this study, would assist in better designing of the septic tank and floating wetland systems to produce effluent quality suitable for disposal or reuse in decentralized areas under stringent guidelines.

The closed-circuit protocols influenced organic and coliform removal; conversely, nutrient and metals removal was independent of circuit connections mode. Aeration appeared to be a potential option to achieve better effluent quality with the employed septic tank (of this study) that might satisfy stricter disposal guidelines. The choice between intermittent and continuous aeration would depend on the influent composition, local discharge guidelines, and funding availability to meet operation and maintenance costs. Intermittent aeration could be an attractive choice as it consumes comparatively less energy compared to continuous aeration, a decisive factor for remote areas. A comparative cost-benefit analysis, particularly on the aeration strategies, could be conducted in future studies to formulate an in-depth knowledge of the financial aspects; such information is necessary for applying the proposed system in decentralized areas.

Kinetic models are an effective tool for predicting the pollutant removal routes in biological systems and designing treatment units. Different models combined with first-order, Monod and multiple Monod-based kinetics were developed to understand pollutant removal routes and the influencing factors in normal and electrode-integrated wetlands (Nguyen et al., 2018; Saeed and Sun, 2011; Saeed et al., 2021c). The pollutant removal pathways and associated influencing factors reported in this study would assist in investigating the reported models or developing new approaches to predict removals in such onsite bioenergy-producing systems and should be focused on in future studies. This study did not analyze the microbial composition of the settled sludge, filler media, and plant roots. Future studies should abridge such a knowledge gap to understand microbial-based pollutant decomposition in decentralized systems comprehensively. This study demonstrates the potential application of electrode-based septic tank-floating wetland systems and the impact of circuit connection and aeration strategies for achieving effluent quality that could be disposed of safely or reused (depending on local guidelines) in decentralized areas.

## 4 Conclusions

The closed-circuit protocol improved the organic removal of the septic tank and floating wetland due to the synergetic impact of electrochemically active and inactive pathways; coliform mortality was also positively influenced by the current flow of the closed-circuit protocol. Nutrient and metals removals were independent of the circuit connection protocol and were primarily achieved by the physicochemical (sedimentation, adsorption, and filtration) and biological (plant uptake and microbial decomposition) pathways. The accumulated sludge and employed media were the major catalysts that supported different removal pathways in the septic tank; the hanging root of the floating wetland primarily contributed to pollutant removal. Intermittent and continuous aeration boosted electrochemically active and inactive microbial decomposition, chemical adsorption, and bioenergy production. Continuous aeration was more effective (compared to intermittent aeration) in terms of nitrogen removal; intermittent aeration further improved organic and coliform removal. Internal resistance was developed in both treatment systems during closed circuit operational protocol. This research developed a novel bioenergy-producing two-stage onsite wastewater treatment system and examined its performance optimizing factors to produce effluent quality suitable for environmental discharge or reuse depending on local discharge guidelines.

## CRediT authorship contribution statement

**Tanveer Saeed:** Writing – original draft, Supervision, Resources, Project administration, Methodology, Investigation, Funding acquisition, Formal analysis, Data curation, Conceptualization. **Abdullah Al-Muyeed:** Writing – review & editing, Funding acquisition. **Takrim Zaman:** Investigation. **Mehedi Hasan:** Investigation. **Tanvir Ahmed:** Writing – review & editing, Funding acquisition.

## Declaration of competing interest

The authors declare that they have no known competing financial interests or personal relationships that could have appeared to influence the work reported in this paper.

## Data Availability

Available in the manuscript
