# Supplementary material for: Bioenergy-producing two-stage septic tank and floating wetland for onsite wastewater treatment: Circuit connection and external aeration
Source: J Environ Manage. 2024 May;359:121011. doi: 10.1016/j.jenvman.2024.121011 (PMC11129191; doi:10.1016/j.jenvman.2024.121011)
Supplement: Multimedia component 1 [file mmc1.docx]

**Supplementary data**

*Supplementary material S1*

**Table.** Probable elemental percentages in the unused and used stone media of the filtration chamber.

| Elements | Unused  (%) | Used  (%) |
| --- | --- | --- |
| C | 3.3 | 11.2 |
| N | 0.2 | 0.3 |
| O | 39.4 | 38.6 |
| Fe | 0.05 | 0.3 |
| Ni | 0.0 | 0.0 |
| Zn | 0.0 | 0.0 |
| Na | 0.05 | 4.1 |
| Mg | 0.6 | 0.4 |
| Al | 13.4 | 9.1 |
| Si | 32.6 | 31.9 |
| P | 0.1 | 0.06 |
| S | 0.0 | 0.0 |
| Pb | 0.1 | 0.1 |
| Cl | 0.1 | 0.1 |
| K | 7.5 | 1.7 |
| Ca | 0.2 | 0.9 |
| Ti | 0.8 | 0.5 |
| Cr | 1.2 | 0.5 |

*Supplementary material S2*







*Unused stone*  *Used stone*

**Figure.** SEM images of the unused and used stone media of the filtration chamber.

*Supplementary material S3*


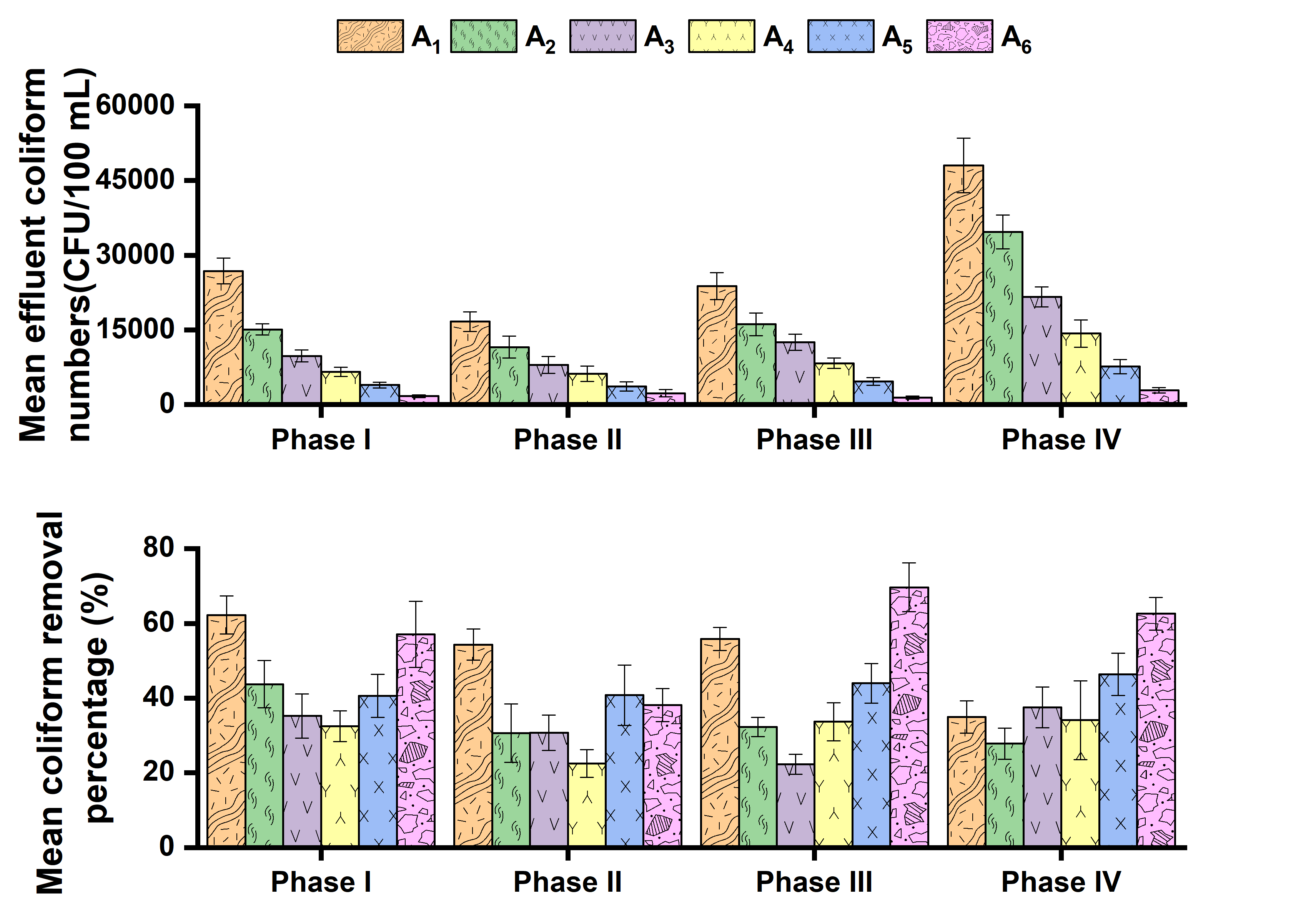


**Figure.** Mean effluent coliform numbers produced in different chambers of the septic tank and the floating wetland, along with associated removal percentages within the four operational phases. Bars indicate standard errors. The symbols A_1_, A_2_, A_3_, A_4_, A_5_, and A_6_ represent wastewater collection points from settling chamber 1, settling chamber 2, the first compartment of the ABR, the second compartment of the ABR, filtration chamber, and floating wetland, respectively.
